# Effects of Delaying Transplanting on Agronomic Traits and Grain Yield of Rice under Mechanical Transplantation Pattern

**DOI:** 10.1371/journal.pone.0123330

**Published:** 2015-04-13

**Authors:** Qihua Liu, Xiu Wu, Jiaqing Ma, Bocong Chen, Caiyun Xin

**Affiliations:** Rice Research Institute, Shandong Academy of Agricultural Sciences, Jinan 250100, Shandong, China; Wageningen University, NETHERLANDS

## Abstract

A delay in the mechanical transplantation (MT) of rice seedlings frequently occurs in Huanghuai wheat-rice rotation cropping districts of China, due to the late harvest of wheat, the poor weather conditions and the insufficiency of transplanters, missing the optimum transplanting time and causing seedlings to age. To identify how delaying transplanting rice affects the agronomic characteristics including the growth duration, photosynthetic productivity and dry matter remobilization efficiency and the grain yield under mechanical transplanting pattern, an experiment with a split-plot design was conducted over two consecutive years. The main plot includes two types of cultivation: mechanical transplanting and artificial transplanting (AT). The subplot comprises four japonica rice cultivars. The results indicate that the rice jointing, booting, heading and maturity stages were postponed under MT when using AT as a control. The tiller occurrence number, dry matter weight per tiller, accumulative dry matter for the population, leaf area index, crop growth rate, photosynthetic potential, and dry matter remobilization efficiency of the leaf under MT significantly decreased compared to those under AT. In contrast, the reduction rate of the leaf area during the heading-maturity stage was markedly enhanced under MT. The numbers of effective panicles and filled grains per panicle and the grain yield significantly decreased under MT. A significant correlation was observed between the dry matter production, remobilization and distribution characteristics and the grain yield. We infer that, as with rice from old seedlings, the decrease in the tiller occurrence, the photosynthetic productivity and the assimilate remobilization efficiency may be important agronomic traits that are responsible for the reduced grain yield under MT.

## Introduction

In China, rice is a staple food, feeding more than 65% of the population. The Huanghuai district is one of the most important rice-production districts contributing to the high grain yield in China. In this region, the rice-wheat rotation is a dominant cropping system with japonica rice as rotation crop after the harvest of wheat, producing approximately 7500 kg of rice grain yield per hectare on average [1]. The conventional cultivation pattern of rice in this region is artificial transplanting, i.e., transplanting rice seedlings into the paddy field by hand, which is very laborious. This cultivation pattern is not only cumbersome but also labor-intensive for farmers, involving working and moving in a stooping posture in a paddy field [2]. In recent years, rural labor has become increasingly insufficient and expensive, mainly due to the migration of labor from the rural areas to the cities. Rural employment decreased from 355 million in 1995 to 314 million in 2007 in China, but the labor ratio of off-farm employment to total employment increased from 33% in 1986 to 63% in 2008 in the Shandong province of China [3,4], triggering enhanced labor costs in agricultural production [5]. The same scenario has also appeared in other developing countries, such as India, Peru, and Bangladesh [6,7,8]. Now, the artificial transplanting mode that cannot meet the requirement of rice production has been gradually replaced due to the serious deficiency in labor employment. Therefore, there is an urgent need to adopt a simple and convenient planting pattern as a substitute to resolve the problem that resulted from labor scarcity and expensive production costs.

The rice mechanical transplanting pattern, which was initially developed in Japan, has been a feasible cultivation pattern and is widely applied in Japan, Korea and other countries due to its ease of cultivation and considerable economic benefits brought by labor cost savings [9,10,11,12]. In China, rice mechanical transplanting technology has been explored since the 1950s. However, until now, the technology has not been widely applied in rice production due to relatively stagnated research based on rice production districts belonging to different cropping systems. In the rice-wheat rotation cropping system, one prominent issue preventing the application of mechanical transplantation technology is the paradox between the optimum rice seedling age at transplanting as required by the transplanting machine and the prolonged seedling age as triggered by some restricting factors [13,14,15]. The optimum rice seedling age, being conducive to the occurrence of tiller, is capable of laying a solid foundation to obtain a high grain yield. In general, young seedlings from 15–20 days of age, being characterized by suitable plant height ranging from 12–17 cm, and stubborn individual plants with enough nutrient supply from the seedling-nursery tray are suitable for mechanical transplanting [16]. These plants take less time to resume normal growth after mechanical harm to roots from being transplanted.

In the Huanghuai district of China, transplanting rice seedlings by machine is frequently delayed, even causing the seedling age to be greater than 40 days, due to the late harvest of wheat, the appearance of unfavorable weather and the limited number of transplanting machines. If sowing is delayed, some varieties with long-growth duration fail to mature normally, thereby inducing the frequent occurrence of the postponement of transplantation. Usually, rice seeds were sown in hard trays at a high density for MT. Because of intensive nutrition competition among individuals, rice seedlings, which grow for a longer duration in hard trays and are not transplanted by machine in time, could receive detrimental influences [13]. These seedlings are customarily called as “old seedlings”. Until now, the performances of agronomy and yield traits in rice from old seedlings under MT remain unclear. Therefore, there is a need to conduct related research with rice plants that are transplanted by hand as controls.

The objective of the study was to test the effects of delaying transplanting on agronomic traits that are related to the grain yield and the yield component of rice under MT. These results will provide useful references for the cultivation of rice via mechanical transplantation in rice-growing districts.

## Materials and Methods

### Ethics statement

We obtained the relevant permission from the corresponding institute (Shandong Rice Research Institute) for planting our materials in the field. This work was unrelated to ethics issues, and no specific permissions were required for the described field study (no specific permissions were required for these locations/activities). We confirm that the field study did not involve endangered or protected species.

### Experimental site and cultivar description

**Fig 1:**
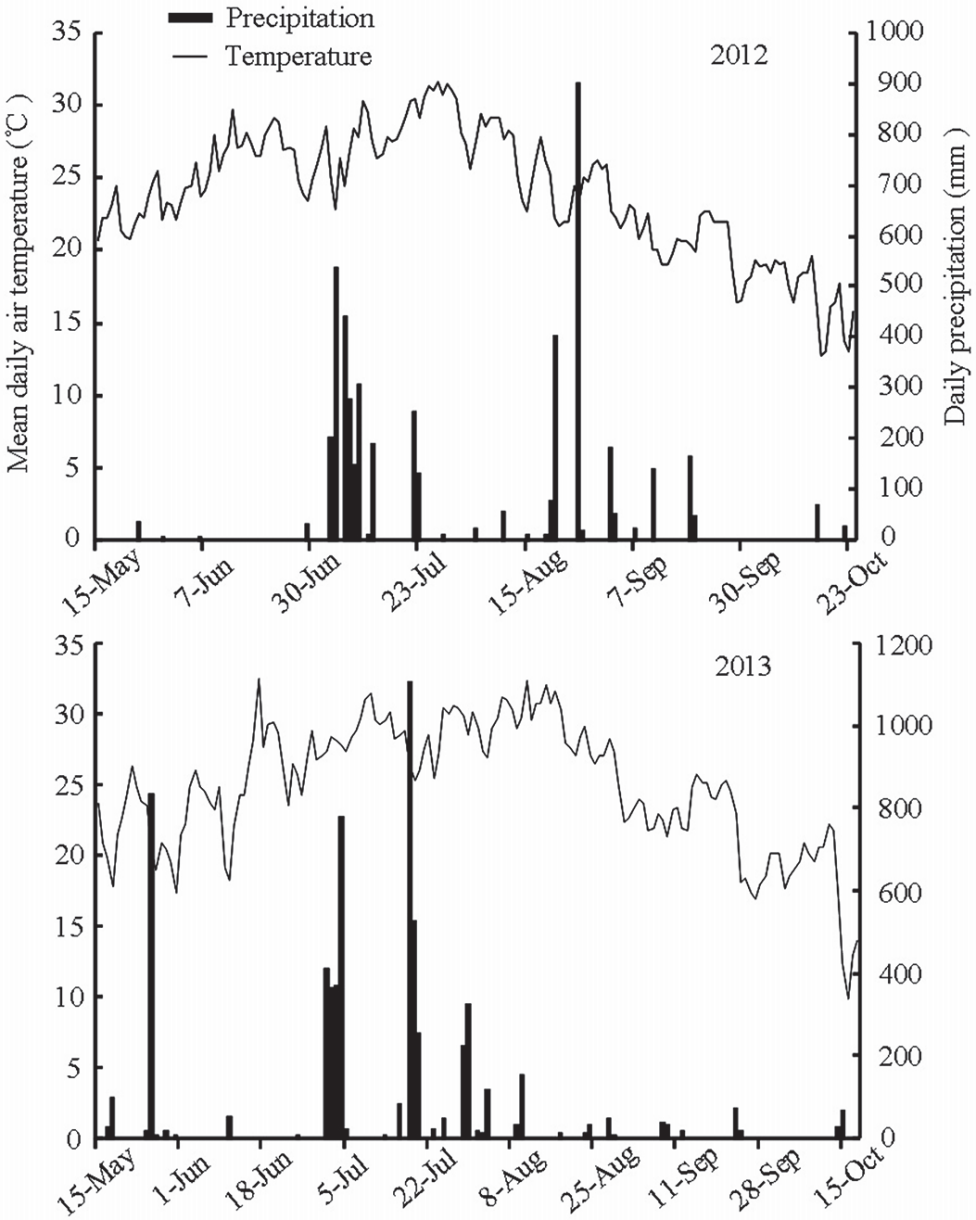
Mean daily air temperature and precipitation during rice growth in 2012 and 2013.

The field experiments were conducted at Yutai (35°00’ N, 116°39’ E) in Shandong province, China, from May to October during 2012 and 2013. The site is located in the Huanghuai district of China, with a cultivation pattern belonging to the typical rice-wheat cropping system. The data of daily average air temperature and rainfall during the rice growth season are shown in Fig 1. In 2012, the means of the daily average air temperature and precipitation were 23.94 °C and 29.09 mm, respectively. In 2013, these values were 24.87 °C and 39.83 mm, respectively. The average temperature in 2013 was approximately 1 °C higher than that in 2012. The total precipitation during the rice growth season in 2012 was 4741 mm, being 1512 mm less than that in 2013. The soil type was clay loam, and the basic fertilities were depicted as follows: organic matter 34.00 g kg^-1^, total nitrogen 1.94 g kg^-1^, available N 74.73 mg kg^-1^, available P 43.98 mg kg^-1^ and available K 199.50 mg kg^-1^. Four conventional *japonica* rice cultivars that are widely cultivated in local production, Shengdao2572, Daliang202, Huaidao11 and Jindao263, were used in the experiment.

### Experimental design and cultivation management

The experiment was a split-plot design with three replications. The transplanting patterns, artificial transplantation (AT) and mechanical transplantation (MT), were assigned as main plots, with four cultivars being randomly allotted to sub-plots. To maintain independent water and fertilizer managements for the two transplanting patterns, the main plots were separated by a 50-cm-wide ridge with plastic film inserted into the soil at a depth of 30 cm.

According to the local rice production, rice seeds were sown on 15 May and 18 May for the artificial and mechanical transplanting patterns, respectively. The ratio of rice seedling bed area to paddy field area was 1:20 under AT while it was 1:80 under MT. The rice seedling nursery bed dimension for each cultivar was 4.5 m^2^ under AT and 1.125 m^2^ under MT. The sowing density was 74.07 g/m^2^ under AT and 411.52 g/m^2^ under MT. The rice seeds were sown on nursery bed for seedling nursery under AT. Urea was broadcast at 7.5 g/m^2^ and 14.9 g/m^2^ when the leaf age reached 2.1 and 4. The soil of nursery bed maintained moisture before the 3-leaf stage and a shallow-water was kept after the 3-leaf stage. The nursery measurements of the rice seedlings for MT were established according to the method of Yu [16] with melioration. Briefly, emerged seeds (150 g) were sown on each plastic tray (the cavity size was 58 cm long by 28 cm wide by 2.8 cm high) containing 4 kg of soil. All of the plastic trays were placed on the nursery bed when the height of the seedlings reached approximately 1.5 cm. Before the leaf age reached 1.1, the seedlings were irrigated and then drained when the water depth was as high as the nursery bed. Subsequently, intermittent irrigation (drying-wetting alternation) was performed, i.e., the seedlings were irrigated until the surface soil in each plastic tray became dry. Urea was applied at 7.5 g/m^2^ when the seedling leaf age was 1.1. The rice seedlings were uniformly transplanted to the paddy field on 28 June for both of the transplanting patterns across the two study years.

When transplanted, the rice seedlings for the mechanical transplantation pattern were 42 days old with an average leaf age of approximately 4.5 and a plant height within 19 cm. A transplanter (KUBATO, SPU-60) that was made in Japan was used to complete the mechanical transplantation. The distances between the rows and hills were 30 cm and 12 cm, respectively, with 3–4 and 4–5 seedlings per hill for the artificial and mechanical transplantation patterns. The size of each sub-plot was 30 m^2^. Fertilizer and other agronomy managements were in accordance with local practices. The total N application amount was 270 kg ha^-1^ with an N, P and K application rate of 2:1:1.

### Sampling and determination

After the rice seedlings were transplanted to the field, the numbers of tiller occurrences of 20 fixed hills from each plot were surveyed at 4-d intervals from 16 to 40 days in 2012 and at 5-d intervals from 11 to 51 days in 2013. The dates of rice jointing, booting, heading and maturity stages in every treatment were recorded, respectively. During the four growth stages that were mentioned above, the above-ground parts of the rice plants from 6 hills from each treatment were sampled and taken to the laboratory, where they were dried at 105 °C for 0.5 h and subsequently divided into two (leaf, and stem and sheath at the jointing stage) or three (leaf; stem and sheath; and panicle at the booting, heading and maturity stages) parts. Then, the separated organs were dried at 70 °C until constant weight and weighed to determine the dry matter weight.

At maturity, thirty plants of each plot were randomly selected to measure the plant height in the field, and the grain yield from each plot was obtained by reaping 3 m^2^ of rice plants (excluding the border plants). The rice grain yield was determined with the moisture content being adjusted to 14%. The agronomic traits of the panicle and yield components, including the panicle length; the number of the primary and secondary branches and the total and filled grains per panicle; the effective number of panicles per m^2^; and the 1000-grain weight, were measured by randomly selecting 30 rice plants (except for the border plants) from each plot.

### Data processing

Based on the parameters that were explicated above, the dry matter remobilization amount, the remobilization efficiency and conversion rate, the reduction rate of the leaf area, the photosynthetic potential and the population growth rate were calculated according to the following equations [17,18]:

Dry matter remobilization amount (g) = Dry matter weight per tiller at heading-dry matter weight per tiller at maturity

Dry matter remobilization efficiency (%) = 100×dry matter remobilization amount/ dry matter weight per tiller at maturity

Dry matter conversion rate (%) = 100×dry matter remobilization amount/dry weight of the grains per tiller at maturity

Leaf area index (LAI) = leaf area of the land area per m^2^

Reduction rate of leaf area (m^2^ d^-1^) = (LAI_2_-LAI_1_) / (t_2_-t_1_)

Where LAI_1_ is the initial LAI, LAI_2_ is the LAI that was measured the second time, t_1_ and t_2_ represent the dates of the first and the second measurements, respectively, and the difference between these dates is the time interval of the two measurements.

Photosynthetic potential of population (m^2^ d) = 1/2(L_1_+L_2_) × (t_2_-t_1_)

Where L_1_ and L_2_ are the leaf areas that were measured at the first and second times, t_1_ and t_2_ represent the dates of the first and the second measurements, respectively, and the difference between these dates is the time interval of the two measurements.

Crop growth rate (g m^-2^ d^-1^) = (w_2_-w_1_) / (t_2_-t_1_)

Where w_1_ and w_2_ represent the dry matter weight of the population tested as measured the first and second times, respectively, and the difference between t_2_ and t_1_ is the time interval of the two measurements.

### Statistical analysis

The data were analyzed by an analysis of variance (ANOVA) using SPSS 11.0 (Statistical Package of the Social Science). To evaluate the differences between the treatments, the means were separated by Least Significance Difference (LSD) at the 5% significance level. The cultivars and cultivation patterns were regarded as fixed factors. The graphics were generated using SigmaPlot 10.0.

## Results

### Performance of the main growth duration for rice from old seedlings under MT

The dates for the main growth stages of the rice under AT and MT are shown in Table 1. In 2012, compared to those under AT, the rice joining, booting, heading and maturity stages were delayed 9–13 d, 7–14 d, 6–11 d and 6–8 d, respectively, under MT. In 2013, these stages were postponed for 3–8 d, 4–5 d, 3–5 d and 4–5 d, respectively. The difference was more pronounced in 2012 than in 2013, with the delayed days for the former being 2–9 d more than those of the latter.

**Table 1:** Dates of the different growth stages of rice under AT and MT in 2012 and 2013.

			Dates for different growth stages			
Year	Cultivar	Cultivation pattern	Jointing	Booting	Heading	Maturity
2012	Shengdao2572	AT	2-Aug	12-Aug	21-Aug	6-Oct
		MT	13-Aug	23-Aug	30-Aug	12-Oct
	Daliang202	AT	4-Aug	16-Aug	25-Aug	13-Oct
		MT	17-Aug	30-Aug	6-Sep	20-Oct
	Huaidao11	AT	9-Aug	22-Aug	1-Sep	15-Oct
		MT	19-Aug	31-Aug	7-Sep	23-Oct
	Jindao263	AT	10-Aug	24-Aug	2-Sep	17-Oct
		MT	19-Aug	1-Sep	8-Sep	24-Oct
2013	Shengdao2572	AT	30-Jul	8-Aug	17-Aug	3-Oct
		MT	3-Aug	13-Aug	22-Aug	7-Oct
	Daliang202	AT	4-Aug	14-Aug	22-Aug	8-Oct
		MT	7-Aug	18-Aug	27-Aug	13-Oct
	Huaidao11	AT	5-Aug	16-Aug	26-Aug	12-Oct
		MT	13-Aug	20-Aug	29-Aug	16-Oct
	Jindao263	AT	6-Aug	17-Aug	26-Aug	13-Oct
		MT	10-Aug	22-Aug	31-Aug	18-Oct

### Dynamics of tiller occurrence for rice from old seedlings under MT

**Fig 2:**
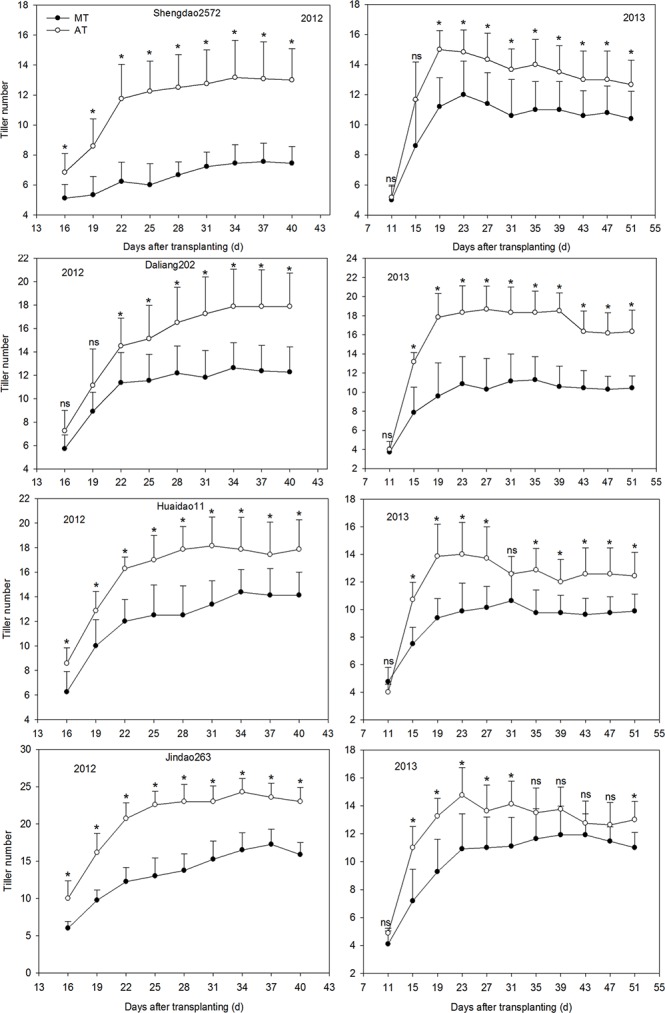
Dynamics of the tiller occurrence under AT and MT in 2012 and 2013. * = *p*≤0.05 and NS = nonsignificant.

The dynamics of tiller occurrence under AT and MT are shown in Fig 2. The tiller number for all of the examined cultivars under MT exhibited a continuous downward trend compared to that under AT in 2012 and 2013. In 2012, compared to that under AT, the tiller numbers under MT decreased by 43.22%, 27.00%, 24.06%, and 35.78%, respectively, in Shengdao2572, Daliang202, Huaidao11 and Jindao263. These values decreased by 20.05%, 39.53%, 23.07%, and 18.80%, respectively, in 2013.

### Above-ground dry matter accumulation traits for rice from old seedlings under MT

As shown in Table 2, the rice dry matter weights (leaf; stem and sheath; and leaf + stem + sheath) at the jointing and booting stages under MT were significantly higher than were those under AT across the two study years, with every variety exhibiting the same change trend. Compared to that under AT, the dry matter weight of the leaf; stem and sheath; and the total above-ground parts at the jointing stage under MT decreased by 31.28%, 17.59% and 25.70% in 2012 and 29.35%, 15.02% and 23.33% in 2013, respectively. Similarly, the corresponding parameters at the booting stage decreased by 24.10%, 18.07% and 21.00% in 2012 and 31.23%, 20.63% and 26.06% in 2013, respectively. In addition, the rice dry matter weights (leaf, stem and sheath, and leaf + stem + sheath + panicle) except for that of the panicle during the heading stages under MT were significantly greater than were those under AT in both years. Unlike the performance of the rice during the above three stages, the dry matter weights of the panicle and the total above-ground parts at maturity exhibited different change tendencies under MT across the two study years. There were obvious varietal differences in the dry matter weight of the leaf, stem and sheath, and panicle, as well as the total above-ground parts, at maturity under MT. In 2012, the difference in the dry matter weight of the leaf between AT and MT was insignificant except for the Shengdao2572 variety. In addition, the dry matter weight of the stem and sheath for Shengdao2572 and Daliang202 significantly decreased, while that of the other varieties did not exhibit a significant change under MT. In 2013, the dry matter weight of the leaf for Daliang202, Huaidao11 and Jindao263 significantly increased under MT, but the opposite occurred for Shengdao2572. For most of the varieties, the dry matter weight of the stem and sheath under MT was not significantly altered excluding Shengdao2572; The dry matter weights of the panicles and the total above-ground parts significantly decreased by 36.79% and 25.69% in 2012 and 29.85% and 19.22% in 2013, respectively.

**Table 2:** Above-ground-part dry matter weight per tiller in rice at the jointing, booting, heading and maturity stages under AT and MT in 2012 and 2013.

			Dry weight at jointing (g tiller^-1^)			Dry weight at booting (g tiller^-1^)			Dry weight at heading (g tiller^-1^)				Dry weight at maturity (g tiller^-1^)			
Year	Cultivar	Cultivation pattern	Leaf	Stem and sheath	Total	Leaf	Stem and sheath	Total	Leaf	Stem and sheath	Panicle	Total	Leaf	Stem and sheath	Panicle	Total
2012	Shengdao2572	AT	0.510a	0.410a	0.920a	0.786a	0.871a	1.656a	0.778a	0.925a	0.395a	2.098a	0.436a	0.868a	1.919a	3.222a
		MT	0.344b	0.344b	0.687b	0.502b	0.588b	1.091b	0.482b	0.719b	0.263b	1.464b	0.346b	0.703b	1.117b	2.165b
	Daliang202	AT	0.469a	0.380a	0.849a	0.879a	0.759a	1.638a	0.886a	1.064a	0.253a	2.202a	0.525a	0.939a	2.013a	3.478a
		MT	0.415b	0.282b	0.697b	0.645b	0.561b	1.206b	0.694b	0.879b	0.319a	1.893b	0.521a	0.806b	1.241b	2.568b
	Huaidao11	AT	0.636a	0.427a	1.063a	0.850a	0.928a	1.778a	0.884a	1.089a	0.370a	2.343a	0.562a	0.965a	2.332a	3.859a
		MT	0.454b	0.386b	0.840b	0.705b	0.864b	1.569b	0.657b	0.984b	0.359a	2.000b	0.547a	0.912a	1.575b	3.034b
	Jindao263	AT	0.821a	0.466a	1.288a	0.767a	0.862a	1.629a	0.804a	1.234a	0.361a	2.399a	0.484a	1.102a	2.489a	4.076a
		MT	0.461b	0.375b	0.837b	0.639b	0.789b	1.428b	0.728b	1.099b	0.356a	2.182b	0.440a	1.067a	1.600b	3.108b
2013	Shengdao2572	AT	0.603a	0.496a	1.099a	0.703a	0.681a	1.384a	0.677a	0.833a	0.366a	1.876a	0.386a	0.784a	1.685a	2.855a
		MT	0.386b	0.474b	0.861b	0.426b	0.448b	0.874b	0.353b	0.506b	0.182b	1.040b	0.259b	0.495b	0.899b	1.652b
	Daliang202	AT	0.575a	0.448a	1.023a	0.831a	0.666a	1.497a	0.792a	0.938a	0.293a	2.023a	0.348b	0.825a	1.799a	2.971a
		MT	0.375b	0.316b	0.690b	0.545b	0.536b	1.081b	0.691b	0.873b	0.326a	1.891b	0.432a	0.793a	1.229b	2.454b
	Huaidao11	AT	0.535a	0.372a	0.907a	0.772a	0.747a	1.518a	0.677a	0.985a	0.296a	1.958a	0.431b	0.879a	2.135a	3.444a
		MT	0.471b	0.348b	0.819b	0.523b	0.626b	1.149b	0.613b	0.921b	0.319a	1.853b	0.532a	0.884a	1.721b	3.138b
	Jindao263	AT	0.662a	0.402a	1.064a	0.867a	0.921a	1.788a	0.683a	0.950a	0.270a	1.903a	0.436b	0.885a	2.180a	3.501a
		MT	0.446b	0.322b	0.768b	0.688b	0.783b	1.471b	0.616b	0.873b	0.295a	1.784b	0.593a	0.858a	1.622b	3.073b

Values of AT and MT for the same cultivar followed by different letters represent significant difference at *p*≤0.05 level.

For every variety, the dry matter accumulation amounts of the rice population under MT were significantly lower than were those under AT across the two study years. Compared to those under AT, the dry matter accumulation amounts of the rice population under MT significantly decreased by 29.62% and 40.27% from the jointing to the booting stage, 50.69% and 35.42% from the booting to the heading stage, and 47.59% and 37.11% from the heading to the maturity stage, respectively, in 2012 and 2013 (Table 3).

**Table 3:** Dry matter accumulative amount of rice population based on three growth stages (from jointing to booting, booting to heading and heading to maturity) under AT and MT in 2012 and 2013.

			From jointing to booting stage	From booting to heading stage	From heading to maturity
Year	Cultivar	Cultivation pattern	Dry matter accumulation (t hm^-2^)	Dry matter accumulation (t hm^-2^)	Dry matter accumulation (t hm^-2^)
2012	Shengdao2572	AT	1.807a	2.426a	4.202a
		MT	0.971b	0.745b	2.108b
	Daliang202	AT	1.276a	2.842a	4.334a
		MT	0.674b	1.651b	2.834b
	Huaidao11	AT	3.449a	3.537a	5.237a
		MT	2.984b	1.213b	2.672b
	Jindao263	AT	3.524a	4.317a	6.425a
		MT	2.448b	2.861b	2.972b
2013	Shengdao2572	AT	2.292a	1.507a	3.742a
		MT	0.614b	0.299b	2.079b
	Daliang202	AT	2.075a	2.596a	3.956a
		MT	1.141b	2.108b	2.667b
	Huaidao11	AT	2.448a	2.666a	4.130a
		MT	1.475b	2.068b	2.620b
	Jindao263	AT	5.054a	1.051a	3.654a
		MT	3.860b	0.575b	2.371b

Values of AT and MT for the same cultivar followed by different letters represent significant difference at *p*≤0.05 level.

### Above-ground dry matter remobilization and distribution characteristics for rice from old seedlings under MT

Compared to those under AT, the leaf dry matter remobilization amount, efficiency and contribution to the grain yield under MT significantly decreased by 55.66%, 38.60% and 29.46% in 2012 and 53.63%, 44.23% and 31.43% in 2013, respectively. The dry matter remobilization amount and the efficiency of the stem and sheath under MT markedly decreased by 55.85% and 57.31% in 2012 and 47.82% and 52.38% in 2013, respectively, compared to those under AT. In terms of the dry matter contribution to the grain of the stem and sheath, the difference between the AT and MT varied with the varieties due to the different genotypes. In 2012, the dry matter contribution of the stem and sheath for Shengdao2572 and Jindao263 under MT significantly decreased, while there was no significant difference between AT and MT for Daliang202 and Huaidao11. In 2013 under MT, this value significantly decreased for most of the varieties excluding Daliang202 (Table 4).

**Table 4:** Above-ground dry matter remobilization characteristics of rice under AT and MT in 2012 and 2013.

			Leaf			Stem and sheath		
Year	Cultivar	Cultivation pattern	Dry matter remobilization amount (g)	Dry matter remobilization efficiency (%)	Dry matter contribution to grain yield (%)	Dry matter remobilization amount (g)	Dry matter remobilization efficiency (%)	Dry matter contribution to grain yield (%)
2012	Shengdao2572	AT	0.342a	44.0a	17.9a	0.0578a	6.251a	3.022a
		MT	0.137b	28.5b	12.2b	0.0166b	2.336b	1.489b
	Daliang202	AT	0.360a	40.8a	18.0a	0.1248a	11.715a	6.229a
		MT	0.173b	25.0b	14.0b	0.0734b	8.343b	5.973a
	Huaidao11	AT	0.322a	36.4a	13.9a	0.1240a	11.403a	5.347a
		MT	0.110b	16.8b	7.1b	0.0717b	7.260b	4.558a
	Jindao263	AT	0.320a	39.7a	13.0a	0.1317a	10.560a	5.274a
		MT	0.176b	28.5b	11.0b	0.0318b	2.895b	1.980b
2013	Shengdao2572	AT	0.291a	43.1a	17.4a	0.049a	5.9a	2.9a
		MT	0.094b	26.4b	10.4b	0.011b	2.1b	1.2b
	Daliang202	AT	0.444a	56.1a	24.7a	0.113a	12.1a	6.4a
		MT	0.259b	37.5b	21.1b	0.081b	9.2b	6.6a
	Huaidao11	AT	0.246a	36.3a	11.5a	0.107a	10.8a	5.0a
		MT	0.081b	13.2b	4.7b	0.036b	3.9b	2.1b
	Jindao263	AT	0.246a	36.1a	11.3a	0.066a	6.9a	3.0a
		MT	0.135b	18.6b	8.3b	0.015b	1.8b	0.9b

Values of AT and MT for the same cultivar followed by different letters represent significant difference at *p*≤0.05 level.

The change trends of the ratios of the leaf, stem and sheath, and panicle dry matter weight to the total weight at the heading stage under MT were different from those under AT across the two study years. The ratio of the leaf dry matter weight to the total weight under MT markedly decreased by 15.13% except for cultivar Jindao263 in 2012, while it decreased by 6.25% except for in Huaidao11 and Jindao263 in 2013. In 2012, the ratio of the dry matter weight of the stem and sheath to the total weight significantly decreased in Shengdao2572 and Huaidao11 under MT but not in the other varieties. However, this difference was insignificant between AT and MT for most of the cultivars in 2013. In 2012, the ratio of the panicle dry matter weight to the total weight under MT significantly increased in Daliang202 and Huaidao11 but showed little change in the other two cultivars. In 2013, this ratio significantly increased in Daliang202, Huaidao11 and Jindao263, while the opposite occurred in Shengdao2572. Compared to AT, the ratios of the leaf, and stem and sheath dry matter weight to the total weight in every variety at maturity under MT significantly increased by 19.56% and 17.52% in 2012 and 27.63% and 10.31% in 2013, respectively. In contrast, the ratio of panicle dry matter weight to the total weight under MT markedly decreased by 15.03% and 12.93% in 2012 and 2013, respectively (Table 5).

**Table 5:** Above-ground dry matter distribution of rice under AT and MT in 2012 and 2013.

			Ratio of the dry weight of the leaf to the total dry weight (%)		Ratio of the dry weight of the stem and sheath to the total dry weight (%)		Ratio of dry weight of the panicle to the total dry weight (%)	
Year	Cultivar	Cultivation pattern	heading stage	Maturity stage	heading stage	Maturity stage	Heading stage	Maturity stage
2012	Shengdao2572	AT	37.1a	13.5b	44.1b	26.9b	18.8a	59.5a
		MT	33.0b	16.0a	49.1a	32.4a	17.9a	51.6b
	Daliang202	AT	40.2a	15.1b	48.4a	27.0b	11.4b	57.9a
		MT	31.7b	20.3a	46.5b	31.5a	16.9a	48.2b
	Huaidao11	AT	37.7a	14.6b	46.5b	25.0b	15.8b	60.4a
		MT	32.9b	18.1a	49.2a	30.2a	17.9a	51.7b
	Jindao263	AT	33.6a	11.9b	51.4a	27.0b	14.9a	61.1a
		MT	33.3a	14.1a	50.4a	34.3a	16.3a	51.5b
2013	Shengdao2572	AT	36.1a	13.5b	44.4b	27.5b	19.5a	59.0a
		MT	33.9b	15.6a	48.4a	29.9a	17.3b	54.5b
	Daliang202	AT	39.1a	11.8b	46.4a	27.8b	14.5b	60.4a
		MT	36.6b	17.6a	46.2a	32.3a	17.3a	50.1b
	Huaidao11	AT	34.6a	12.5b	50.3a	25.5b	15.1b	62.0a
		MT	33.1a	17.0a	49.7a	28.2a	17.2a	54.8b
	Jindao263	AT	35.9a	12.5b	49.9a	25.3b	14.2b	62.2a
		MT	34.5a	19.3a	49.0a	27.9a	16.5a	52.7b

Values of AT and MT for the same cultivar followed by different letters represent significant difference at *p*≤0.05 level.

### Population photosynthetic ability and growth rate for rice from old seedlings under MT

Compared to that under AT, leaf area index at the jointing, booting, heading and maturity stages under MT significantly decreased by 48.05%, 35.36%, 37.11% and 58.01% in 2012 and 48.55%, 35.76%, 32.58% and 57.54% in 2013, respectively; The reduction rate of the leaf area from heading to maturity under MT significantly decreased by 10.35% in 2012 and 7.92% in 2013, respectively. In 2012, the photosynthetic potential and crop growth rate under MT significantly decreased by 43.31% and 25.49% from jointing to booting, 51.81% and 34.51% from booting to heading, and 47.06% and 46.66% from heading to maturity. In 2013, these values were 40.07% and 39.78% from jointing to booting, 37.52% and 35.92% from booting to heading, and 42.18% and 37.17% from heading to maturity, respectively (Table 6).

**Table 6:** Leaf area index, reduction rate of the leaf area, photosynthetic potential and crop growth rate of rice under AT and MT in 2012 and 2013.

			Leaf area index				Reduction rate of leaf area (LAI d^-1^)	Photosynthetic potential (m^2^ d)			Crop growth rate (g m^-2^ d^-1^)		
Year	Cultivar	Cultivation pattern	Jointing	Booting	Heading	Maturity	from heading to maturity	From jointing to booting	From booting to heading	From heading to maturity	From jointing to booting	From booting to heading	From heading to maturity
2012	Shengdao2572	AT	4.015a	6.529a	6.389a	3.933a	0.052b	57.991a	64.593a	242.604a	16.419a	24.252a	8.936a
		MT	2.204b	4.152b	4.140b	1.711b	0.057a	34.960b	33.168b	125.806b	8.819b	9.306b	4.899b
	Daliang202	AT	4.558a	6.160a	7.203a	4.392a	0.057b	69.669a	66.815a	284.082a	9.812a	28.405a	8.841a
		MT	2.338b	4.251b	4.749b	2.032b	0.062a	46.124b	31.502b	149.199b	4.809b	23.580b	6.276b
	Huaidao11	AT	5.137a	6.661a	7.250a	5.221a	0.046b	82.582a	76.506a	274.341a	24.623a	32.133a	11.897a
		MT	2.425b	4.052b	4.144b	1.798b	0.051a	42.100b	32.784b	136.672b	22.941b	15.155b	5.806b
	Jindao263	AT	5.632a	7.949a	7.775a	5.616a	0.048b	101.857a	78.618a	301.296a	23.484a	43.144a	14.270a
		MT	3.081b	5.190b	4.965b	2.506b	0.054a	53.762b	40.621b	171.844b	18.824b	35.746b	6.458b
2013	Shengdao2572	AT	3.725a	5.849a	5.579a	3.333a	0.047b	38.296a	51.426a	213.890a	22.913a	16.740a	7.791a
		MT	1.977b	3.836b	3.856b	1.396b	0.052a	29.068b	34.614b	123.417b	6.132b	3.321b	4.421b
	Daliang202	AT	4.815a	7.559a	7.226a	4.765a	0.052b	61.869a	59.299a	288.741a	20.742a	32.437a	8.238a
		MT	1.962b	4.778b	4.531b	1.847b	0.056a	37.069b	41.891b	153.073b	10.370b	23.404b	5.553b
	Huaidao11	AT	5.075a	6.953a	6.282a	3.826a	0.051b	66.156a	66.174a	242.593a	22.241a	26.647a	8.599a
		MT	3.108b	4.127b	4.168b	1.522b	0.054a	32.556b	29.030b	139.392b	16.381b	22.965b	5.344b
	Jindao263	AT	5.239a	7.195a	6.718a	4.193a	0.052b	74.605a	62.610a	267.321a	42.093a	11.675a	7.452a
		MT	2.654b	4.961b	4.843b	2.078b	0.056a	45.691b	44.119b	169.566b	32.153b	6.381b	4.837b

Values of AT and MT for the same cultivar followed by different letters represent significant difference at *p*≤0.05 level.

### Performance of the agronomic traits and grain yield for rice from old seedlings under MT

**Fig 3:**
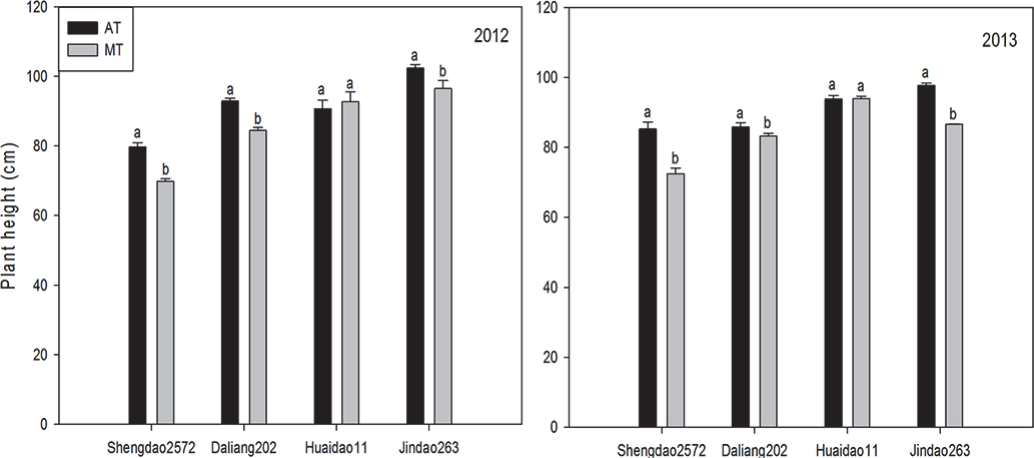
Rice plant height at maturity under AT and MT in 2012 and 2013. Values of AT and MT for the same cultivar followed by different letters represent significant difference at *p*≤0.05 level.

As shown in Fig 3, a significant difference in the plant height at maturity between AT and MT was found. Except for cultivar Huaidao11, the plant height for the other cultivars under MT was 8.79% less than that under AT in 2012. Similarly, the plant height significantly decreased by 9.85% in 2013.

Compared to AT, there was a significant decrease in the panicle length under MT in 2013; however, the difference between both of the treatments was not significant except for that in cultivar Shengdao2572 in 2012. The number of primary branches per panicle markedly decreased by 11.12% and 15.65% under MT, except for Huaidao11 in 2012 and Daliang202 and Huaidao11 in 2013. Under MT, the number of the secondary branches per panicle significantly decreased by 36.10% and 27.56%, excluding Shengdao2572 and Huaidao11 in 2012 and Huaidao11 in 2013. In 2012, the seed-setting rate markedly decreased except for that in Daliang202 under MT compared to that under AT. Nevertheless, no significant difference between the two treatments was observed except for in Shengdao2572 in 2013. There was a difference in the 1000-grain weight among the cultivars and study years between AT and MT. In 2012, the 1000-grain weight of Shengdao2572 under MT significantly decreased, while the opposite occurred for Jindao263, with little alteration being found in Daliang202 and Huaidao11. In 2013, the 1000-grain weight significantly increased under MT in Shengdao2572 and Jindao263 but not in the other two cultivars. The effective panicles per mm^2^, total grains per panicle (except Huaidao11) and grain yield significantly decreased by 28.97%, 18.34% and 31.26%, respectively, in both of the study years (Table 7).

**Table 7:** Agronomic traits of the panicle, grain yield and yield components in rice under AT and MT in 2012 and 2013.

Year	Cultivar	Cultivation Pattern	Panicle length (cm)	No.of the primary branch per panicle	No. of the secondary branch per panicle	No. of effective panicles m^-2^	No. of total grainspanicle^-1^	Seed-setting rate (%)	1000-grain weight (g)	Grain yield(kg m^-2^)
2012	Shengdao2572	AT	16.45a	10.73a	19.57a	380.80a	98.13a	0.89a	26.72a	0.66a
		MT	14.64b	9.70b	17.17a	265.72b	78.45b	0.77b	25.73b	0.46b
	Daliang202	AT	16.02a	12.30a	26.82a	438.76a	120.96a	0.93a	25.97a	0.97a
		MT	15.89a	10.93b	17.70b	358.68b	95.45b	0.90a	26.50a	0.62b
	Huaidao11	AT	16.26a	11.51a	17.36a	396.76a	109.94a	0.91a	27.20a	1.10a
		MT	16.40a	11.50a	17.90a	242.76b	106.79a	0.77b	27.37a	0.60b
	Jindao263	AT	16.09a	12.31a	20.71a	620.76a	110.82a	0.95a	25.55b	1.17a
		MT	16.40a	10.78b	12.67b	294.00b	99.99b	0.84b	27.27a	0.80b
2013	Shengdao2572	AT	18.23a	11.67a	22.39a	346.92a	131.11a	0.78a	23.64b	0.80a
		MT	16.65b	10.55b	19.40b	263.76b	109.00b	0.73b	27.12a	0.43b
	Daliang202	AT	17.14a	11.67a	25.90a	399.00a	118.27a	0.90a	25.69a	1.25a
		MT	14.75b	11.10a	18.91b	317.24b	105.86b	0.93a	25.68a	0.93b
	Huaidao11	AT	17.97a	11.43a	21.00a	406.00a	120.61a	0.91a	28.29a	0.94a
		MT	16.60b	10.72a	21.20a	284.76b	123.78a	0.90a	27.46b	0.82b
	Jindao263	AT	17.85a	12.93a	23.37a	426.16a	145.28a	0.89a	26.50b	1.14a
		MT	15.57b	10.20b	13.60b	399.00b	102.93b	0.87a	27.15a	0.86b

Values of AT and MT for the same cultivar followed by different letters represent significant difference at *p*≤0.05 level.

### Relationships between the grain yield and dry matter production, remobilization and distribution characteristics

**Fig 4:**
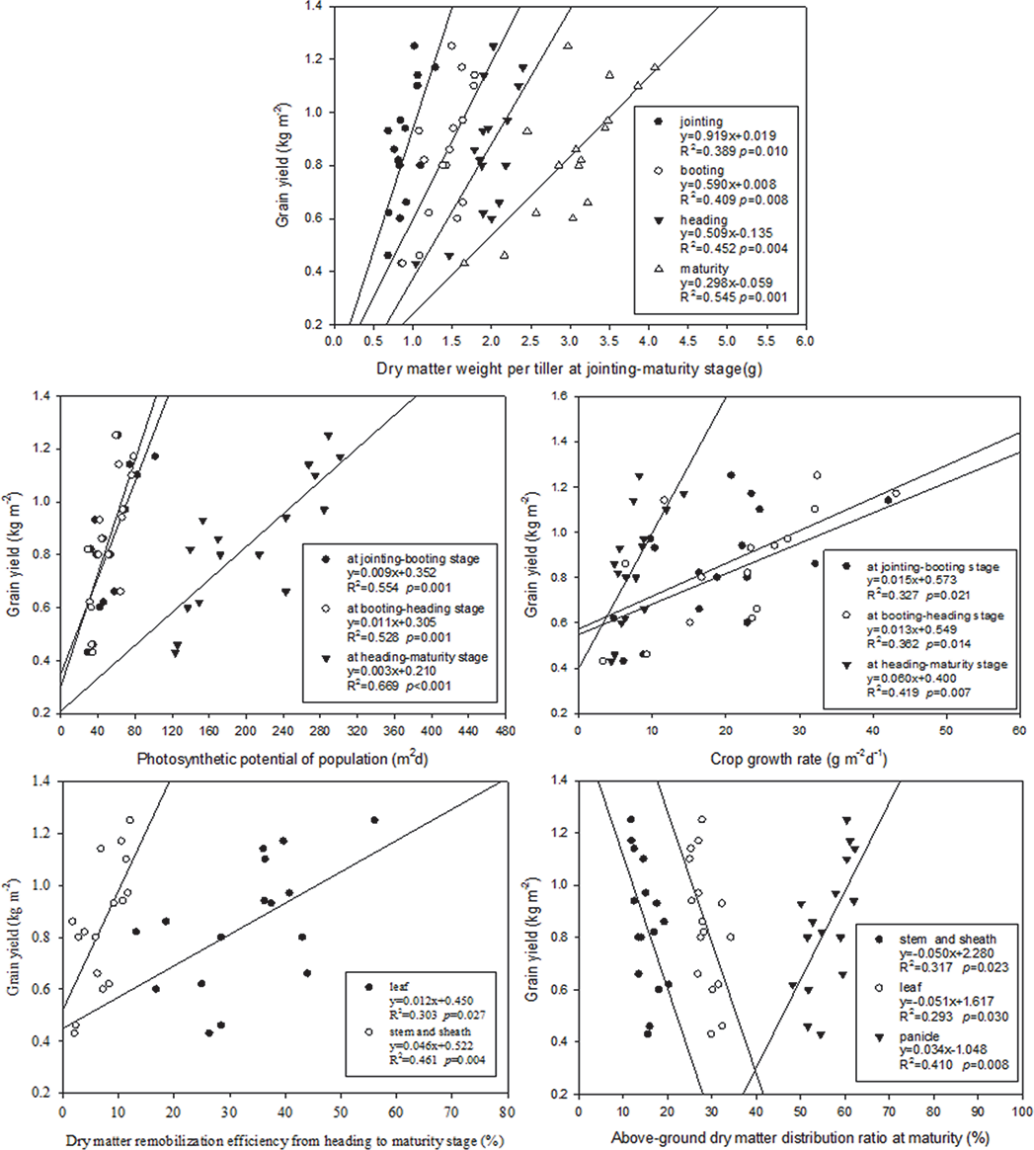
Relationship between grain yield and the dry matter per tiller, the photosynthetic potential of the population, the crop growth rate, the dry matter remobilization efficiency and the above-ground dry matter distribution ratio at maturity.

There existed a significant and positive correlation between the dry matter weight per tiller at the jointing-maturity stage, the population photosynthetic potential, the crop growth rate, the dry matter remobilization efficiency at the heading-maturity stage, the panicle dry matter distribution ratio at maturity and the grain yield. Furthermore, there was a significant and negative correlation between the leaf dry matter distribution ratio and the stem and sheath dry matter distribution ratio and grain yield. These relationships were well simulated by linear equations, which were presented in both of the study years (Fig 4).

## Discussion

Delayed rice growth stages under MT compared to under AT were previously reported by Huo [19], who demonstrated that the rice-jointing, heading and maturity stages were delayed by 5–8 d, 2–3 d, and 1–2 d, respectively, when the rice seedlings were transplanted at the optimum seedling age (approximately 20 d). This study demonstrates that, compared to those under AT, rice jointing, booting, heading and maturity stages were remarkably delayed by 8–12 d, 6–13 d, 5–9 d and 5–6 d under MT in 2012, respectively, while they only had a marginal change in magnitude with a delay of 2–7 d under MT in 2013. The difference between both of the study years can be explained as higher air temperature during rice growth in 2013, especially from the booting stage to the heading stage (Table 1 and Fig 1). The delayed rice growth under MT may be attributable to the deleterious effects on the roots of mechanical transplantation. In general, it takes rice plants approximately 10–14 d to recover from injury. The age of the seedlings at transplantation significantly affects the number of productive tillers [20]. When rice seedlings are transplanted at the right time in terms of age, tillering and growth proceed normally [21]. The occurrence of tillering for rice with 20-d-old seedlings under MT commenced from the third leaf position after the seedlings were moved into a paddy field, and the peak number of tillers was much greater under MT than that under AT [22]. In contrast to those under AT, the rice tillers under MT occurred 7–10 d later but increased more rapidly once normal growth resumed after being transplanted [22]. In the experiment, the occurrence of rice tiller under MT began almost from the 12th day after being transplanted. It is noteworthy that the maximum and increasing rates of rice tillers under MT were markedly smaller than were those under AT, which is inconsistent with the report of Li [22]. This contradiction may be ascribed to the intensive competition among individual seedlings under the limited growth conditions of the seed-nursery bed under MT, which lasts longer for old seedlings than that for normal seedlings with optimum seedling age [21]. As a result, the tiller occurrence in rice from old seedlings under MT was severely hampered by the delay in transplantation, i.e., the later the rice seedlings were transplanted by machine, the more severely was hindered the rice tiller growth.

The above-ground dry matter weights per tiller and population are pivotal parameters reflecting the growth potential of individuals and populations of rice. The population dry matter weight is determined by the tiller number and dry matter accumulative amount per tiller. Therefore, realizing the growth potential of individuals is beneficial to the improvement of the ability to produce dry matter for the rice population. A previous study reported that the dry matter weight per tiller for mechanically transplanted 20-d-old rice seedlings was 13.15%, 7.73%, 8.77% and 10.84% lower than that of rice that was transplanted by hand at the jointing, booting, heading and maturity stages, respectively [24]. In terms of the dry matter accumulative amount of the population, significant differences between MT and AT were detected, with the former being 6.95% and 11.00% lower than the latter from jointing to heading and from heading to maturity [23]. In the present study, the dry matter weight of the leaf or the stem and sheath from jointing to maturity as well as that of the panicle from heading to maturity in rice (seedling age 42 d) under MT significantly declined compared to that under AT. In addition, a similar tendency also occurred in the dry matter accumulative amount of the rice population from jointing to booting, from booting to heading and from heading to maturity (Tables 1 and 2), which seems to be similar to the reported results [23]. Nevertheless, the differences between AT and MT expanded in our study compared to those in the above-mentioned report, suggesting that the growth and development of rice plants under MT was evidently retarded with the delayed transplantation.

In general, rice grain-filling matter consists of nonstructural carbohydrates that are stored in the stem, sheath and leaves pre-anthesis and of photosynthetic products created mainly by leaves post-anthesis, with the former accounting for nearly 30% [23]. Regarding the rice plants that were transplanted by machine at a 20-d seedling age, the dry matter remobilization amount and efficiency and the contribution rate to the leaf yield decreased by 21.41%, 13.16% and 14.35%, respectively, whereas these values increased by 11.06%, 20.48% and 21.16% for the stem and sheath compared to rice plants that were transplanted by hand [23]. According to the data of the present study, under MT, the above-mentioned parameters (except for the contribution rate to the yield for the stem and sheath) were significantly lower than were those under AT (Table 4). These results indicate that the supply of photosynthetic assimilates from source (leaf + stem and sheath) to sink (grain) is lowered for rice plants from old seedlings under MT, which may be linked to decreased sink capacity (Table 7). The photosynthetic potential is a key index representing the photosynthesis productivity of the crop population, which depends on both the leaf index and the duration of leaf photosynthesis [25]. The growth rate of a crop population, a parameter embodying the dry matter production per day, describes the efficiency of photosynthesis productivity [26]. Our results demonstrate that, in contrast to those under AT, the rice photosynthetic potential and the population growth rate at the jointing-heading and heading-maturity stages significantly decreased under MT (Table 6). The differences between AT and MT are greater than those that were reported by Li [23], who found that under MT, the rice (20-d seedling age) photosynthetic potential at every growth stage was 11.19% lower than that under AT, while the population growth rate was slightly lower than that under AT from the jointing to the heading stage (with a significant decrease only from heading to maturity). One possible explanation for the difference in these agronomic traits between AT and MT is the shortage of vegetative growth due to the inhibited growth in the nursery bed and the postponed recovery from mechanical transplantation injury for mechanically transplanted rice, which was also evidenced by the decreased plant height and tiller occurrence (Figs 2 and 3). Furthermore, if the rice plants are mechanically transplanted after the appropriate seedling age, the vegetative growth duration of rice is further shortened due mainly to the longer growth inhibition in the nursery bed, which demonstrates that the development of the tiller, the photosynthetic production and the assimilate remobilization efficiency in rice from old seedlings are severely impaired. These speculations demonstrate that the above-ground characteristics of rice plants after transplanting varied with seedling age.

Seedling age is an important element affecting the number of filled grains per panicle, the panicle length, the 1000-grain weight and the grain yield in rice, as reported by Ginigaddara [20]. The timely transplantation and appropriate seedling age are essential for producing a higher grain yield, while the seedling age depends on many factors, including crop rotation, climate condition, etc., and might be different in different production systems [27,28,29,30]. Li [23] reported that, when rice plants were mechanically transplanted at a 20-d seedling age, the grain yield markedly decreased by 4.66% compared to that under AT. However, in the present study, the rice (42-d seedling age) grain yield under MT was 31.26% lower than that of AT (Table 7). These results indicate that delaying transplanting has a significant negative influence on the rice grain yield under MT. In addition, our results indicate that the number of effective panicles per m^2^ under MT significantly decreased compared to that under AT, and a similar trend was observed in the number of primary and secondary branches and the total grains per panicle (except for in the cultivar Huaidao11) (Table 7). The significant decrease in the number of effective panicles is largely attributed to poorer tiller occurrence under MT (Fig 2). Therefore, we argue that the significant reduction in the number of effective panicles and filled grains per panicle is the direct cause of the decreased grain yield under MT. This study reported that high-yielding rice was characterized by great population dry matter weight and photosynthetic potential and a slowed leaf-area reduction rate. After the heading stage, transferring more photosynthetic product from being stored in the leaf, stem and sheath to the panicle allows rice to obtain a high grain yield; in other words, a higher dry matter distribution ratio of the panicle is instrumental in increasing the grain yield. Our results indicate that dry matter weight per tiller at the jointing-maturity stage, the population photosynthetic potential, the crop growth rate, the dry matter remobilization efficiency and the panicle dry matter distribution ratio are significantly and positively correlated with grain yield, while the leaf dry matter distribution ratio and the stem and sheath dry matter distribution ratio are significantly and negatively related with the grain yield (Fig 4). Therefore, we speculate that the decreased photosynthetic potential, population growth rate, dry matter accumulative amount, dry matter distribution ratio of the panicle and accelerated reduction of the leaf area are important physiological factors in the decreased rice grain yield under MT. Interestingly, compared to that under AT, the 1000-grain weight of cultivar Jindao263 under MT significantly increased irrespective of the impaired photosynthetic productivity and assimilate supply. Presumably, the sink capacity (effective panicles and filled grains) decreases more than does the source supply (photosynthetic productivity); in other words, the source-sink ratio increases, thereby leading to an increased grain weight in Jindao263.

Compared with AT, the tiller numbers, dry matter weight, dry matter accumulation, LAI, growth rate, photosynthetic potential, dry matter remobilization efficiency of leaf under MT were significantly decreased under MT. Those agronomic traits above are attributed to grain yield reduced under MT. Taken together, delaying transplanting severely restricted rice growth and grain yield under MT in the Huanghuai region of China. Consequently, it is crucial to adopt effective measurements to mitigate the phenomenon. Firstly, altering the settings of the transplantation machine to lessen damage during transplantation may be an alternative in shortening resuming growth period and expediting rice seedling tillering. Secondly, lowering sowing density could decrease the competition among plant individuals in nursery bed and improve rice seedling quality. Improving the settings of the transplantation machine could match the lowered sowing density. Thirdly, screening varieties with relative insensitivity to delaying transplanting and consistent performance in yield traits may be also an effective channel.

## Conclusions

This investigation indicates that using old rice seedlings would result in postponed growth stages, poor tiller occurrence, limited dry matter accumulation and lower population growth rate, photosynthetic potential, and grain yield under MT. Compared to those under AT, the tiller number, population photosynthetic productivity, and dry matter remobilization efficiency significantly decreased under MT due primarily to the obviously short vegetative growth duration, which is the main physiological reason for the decreased grain yield. The significant decreases in the filled grains per panicle and in the number of effective panicles in particular directly led to a decreased rice grain yield, especially that of effective panicles.
